# Oxygen Stewardship in Adult Critical Care: A Quality Improvement Initiative

**DOI:** 10.1111/nicc.70476

**Published:** 2026-04-06

**Authors:** Wei Jun Dan Ong, Glen JiaXin Li, Lawrence Matunan Ace Azul, Anna Kristina Trajico, Wee Kian Ricardo Tan, Jared D'Souza, Faheem Ahmed Khan, Amit Kansal

**Affiliations:** ^1^ College of Health Sciences Rush University Chicago Illinois USA; ^2^ Department of Respiratory Therapy Ng Teng Fong General Hospital, National University Health System Singapore Singapore; ^3^ Department of Intensive Care Unit Nursing Ng Teng Fong General Hospital, National University Health System Singapore Singapore; ^4^ Department of Intensive Care Medicine Ng Teng Fong General Hospital, National University Health System Singapore Singapore

**Keywords:** clinical compliance, critical care nursing, oxygen stewardship, oxygen therapy, quality improvement

## Abstract

**Background:**

Oxygen therapy is commonly administered in critical care, yet patients frequently receive oxygen above recommended saturation targets. Excessive oxygen exposure may contribute to avoidable harm and unnecessary resource use. Despite guideline recommendations, reliable adherence to oxygen titration targets in routine intensive care practice remains inconsistent.

**Aim:**

To evaluate the impact of a nurse‐engaged oxygen stewardship quality improvement initiative on adherence to oxygen saturation targets and oxygen consumption in an adult intensive care unit.

**Study Design:**

A clinician‐led, before‐and‐after quality improvement evaluation was conducted over 21 months in a mixed adult intensive care unit. The intervention integrated a standardised oxygen weaning protocol, multidisciplinary education, audit and feedback through spot checks, workflow reminders and iterative refinement informed by staff surveys and interviews.

**Results:**

The proportion of patient‐days outside target oxygen saturation ranges decreased from a median of 63% (IQR 57–65) pre‐intervention (*n* = 2686 patient‐days) to 20% (IQR 15–22) post‐intervention (*n* = 5621 patient‐days), with sustained improvement over 12 months. Median monthly oxygen consumption per patient‐day declined significantly for conventional oxygen therapy, non‐invasive ventilation and mechanical ventilation. High‐flow nasal cannula therapy remained associated with higher non‐compliance rates and showed no statistically significant reduction in oxygen consumption. Reductions in overall oxygen use were primarily attributable to decreased oxygen delivery during non‐compliant periods, while oxygen use within target ranges remained stable.

**Conclusions:**

Embedding oxygen stewardship into routine critical care workflows was associated with sustained improvements in oxygen titration practice and reduced oxygen consumption. Behavioural reinforcement and integration into daily nursing practice were key to maintaining adherence.

**Relevance to Clinical Practice:**

Structured oxygen stewardship initiatives can support critical care nurses in delivering guideline‐adherent oxygen therapy, strengthen accountability during handovers and reduce unnecessary oxygen exposure without compromising appropriate treatment.

## Impact Statements

- What is known
  ○ Oxygen therapy is frequently administered above recommended saturation targets in ICU practice.
  ○ Excessive oxygen exposure may contribute to patient harm and unnecessary resource use.
  ○ Previous studies suggest that education and guideline dissemination alone are insufficient to sustain practice change.
- What this paper adds
  ○ A structured oxygen stewardship programme improved adherence to oxygen saturation targets in a 34‐bed ICU.
  ○ Sustained reductions in oxygen consumption were achieved alongside no identified increase in adverse respiratory events during routine clinical monitoring.
  ○ Embedding oxygen titration into nursing workflows and audit feedback mechanisms supported durable behavioural change.

## 1 Introduction

Oxygen therapy is widely used in acute and critical care to treat hypoxaemia and respiratory failure [1]. However, oxygen is frequently administered above recommended targets or continued longer than clinically necessary [2]. Excessive oxygen exposure may contribute to oxidative stress and delayed recovery [3], while unnecessary oxygen use increases healthcare resource utilisation [4]. In intensive care settings, oxygen titration is often nurse‐led, positioning critical care nurses at the centre of safe and effective oxygen stewardship.

International guidelines recommend maintaining oxygen saturation within defined target ranges, typically 92%–96% for most adults and 88%–92% for those at risk of hypercapnia [5, 6]. Despite these recommendations, adherence in routine clinical practice remains inconsistent. Studies report persistent liberal oxygen use due to delayed reassessment, unclear documentation, competing priorities and safety concerns among clinicians [7, 8].

In the ICU, this gap between evidence and practice represents both a clinical and quality issue. Oxygen prescribing and weaning occur across shifts and professional groups and depend heavily on communication, workflow integration and shared accountability [9]. Improving oxygen titration, therefore, requires more than guideline dissemination; it requires structured quality improvement strategies embedded into daily practice [10, 11].

This study evaluated the impact of a structured oxygen stewardship quality improvement programme in a tertiary adult ICU. The intervention integrated a standardised oxygen weaning protocol aligned with guideline‐recommended targets, multidisciplinary education and iterative reinforcement through audit and feedback. Using routinely collected clinical data, we examined changes in oxygen compliance and oxygen consumption before and after implementation. By framing oxygen stewardship as a systems‐based quality initiative, this study aims to provide practical, transferable insights to strengthen oxygen titration practices in critical care.

## 2 Methods

### 2.1 Study Design and Setting

We conducted a clinician‐led, before‐and‐after quality improvement evaluation in a 34‐bed mixed‐adult ICU at a tertiary public hospital. The ICU admits medical and surgical patients requiring advanced respiratory and organ support. Oxygen titration decisions were primarily undertaken by bedside critical care nurses in collaboration with respiratory therapists and physicians.

The evaluation spanned 21 months (April 2024 to December 2025) and comprised three phases: a pre‐intervention period (April to September 2024), a transition and implementation phase (October to December 2024) and a post‐intervention period (January to December 2025). All adult patients receiving supplemental oxygen via conventional oxygen therapy, non‐invasive ventilation (NIV), mechanical ventilation (MV) or high‐flow nasal cannula (HFNC) during the study period were eligible for inclusion.

Prior to the intervention, oxygen therapy was managed according to general ICU practice and international guideline recommendations. No unit‐wide oxygen stewardship protocol, structured education programme or bedside decision‐support tools were formally implemented. No formal exclusion criteria were applied. Patients receiving end‐of‐life care, palliative extubation or comfort‐focused management were included in the evaluation because oxygen titration decisions remained part of routine bedside care.

The project was reviewed by the institutional review board (DSRB reference number 2024‐3636; approval granted: 20 August 2024) and a waiver of informed consent was granted as data were de‐identified and collected as part of routine quality monitoring.

### 2.2 Intervention

The oxygen stewardship initiative was implemented using iterative Plan‐Do‐Study‐Act (PDSA) cycles.

The first phase introduced a standardised oxygen weaning protocol aligned with guideline‐recommended oxygen saturation targets (92%–96% for most adults; 88%–92% for patients at risk of hypercapnia). Multidisciplinary education sessions were conducted to raise awareness of hyperoxia risks, reinforce appropriate titration practices and clarify documentation expectations. Education targeted nurses, respiratory therapists and physicians, with emphasis on bedside titration and interprofessional communication.

Subsequent PDSA cycles incorporated structured audit and feedback mechanisms. Targeted spot checks were conducted at predefined times during routine ICU workflow (morning nursing handover, post‐ward rounds and evening handover) to assess adherence to oxygen saturation targets and documentation of oxygen plans. Observations were used for real‐time, non‐punitive feedback to reinforce appropriate titration behaviour. Spot checks and feedback were conducted by respiratory therapists and senior ICU Green ambassador nurses involved in the stewardship initiative, with findings communicated during routine clinical interactions and handovers.

Findings from an anonymous cross‐sectional staff survey informed additional refinements. The survey assessed knowledge, perceived barriers and attitudes towards oxygen stewardship. Semi‐structured interviews with ICU nurses, respiratory therapists and physicians were subsequently conducted to explore persistent challenges in oxygen titration and protocol implementation. Interviews followed a brief topic guide focusing on barriers, facilitators and workflow considerations related to oxygen weaning. Responses were summarised using a pragmatic thematic approach to identify recurring implementation challenges.

### 2.3 Study of the Intervention

The primary objective of the evaluation was to assess changes in adherence to target oxygen saturation ranges and oxygen consumption before and after implementation of the stewardship initiative. Clinical endpoints such as ICU length of stay and mortality were not prespecified outcomes, as the focus was on process reliability and resource utilisation.

Monthly compliance rates and oxygen consumption were monitored throughout the post‐intervention period to assess the sustainability of practice change.

### 2.4 Measures

#### 2.4.1 Compliance With Oxygen Targets

The primary process measure was adherence to prescribed oxygen saturation targets. Peripheral oxygen saturation (SpO_2_) values were assessed at three predefined time points daily (08:00, 14:00 and 22:00), corresponding to routine nursing handovers and ward rounds. These time points were selected pragmatically to reflect steady clinical states following major care transitions.

A patient‐day was classified as non‐compliant if SpO_2_ exceeded the upper target limit at the scheduled assessment without a corresponding adjustment in oxygen flow or the fraction of inspired oxygen (FiO_2_). This operational definition was chosen to reflect sustained deviation from recommended practice rather than transient fluctuations. Target saturation ranges were consistently applied across all oxygen delivery modalities, unless clinically contraindicated.

Patients breathing room air without supplemental oxygen were included in the denominator for patient‐days but were not classified as non‐compliant if SpO_2_ values exceeded the upper target limit, as oxygen therapy could not be further reduced. These observations were therefore considered clinically acceptable and excluded from the non‐compliance classification.

Compliance rates were calculated as the proportion of compliant patient‐days relative to total eligible patient‐days and were analysed overall and stratified by oxygen delivery modality.

#### 2.4.2 Oxygen Consumption

Oxygen consumption was estimated for each patient‐day using documented oxygen flow rates, FiO_2_ settings and duration of therapy. Estimates were time‐weighted based on recorded values at predefined assessment points, with settings assumed to remain constant between observations. Modality‐specific equations were applied (Table S1).

Total estimated oxygen volume was calculated per patient‐day and aggregated by study phase and oxygen delivery modality. Oxygen use was additionally analysed according to compliance status to examine associations between adherence and resource utilisation.

### 2.5 Data Collection and Governance

Patient‐level data were extracted from the electronic medical record in de‐identified form and analysed in aggregate. Spot‐check observations and staff feedback activities were conducted as part of routine quality assurance. Staff participation in surveys and interviews was voluntary, responses were anonymised and findings were reported at group level only.

Reporting of this initiative was guided by the SQUIRE 2.0 framework for quality improvement studies.

### 2.6 Statistical Analysis

Descriptive statistics were used to summarise compliance rates and oxygen consumption across study phases. Continuous variables were reported as medians with interquartile ranges. The Mann–Whitney U test was used to compare median oxygen consumption between compliant and non‐compliant patient‐days.

Monthly trends in compliance and oxygen consumption were examined descriptively to assess the sustainability of practice change. Interrupted time‐series modelling was not undertaken, as the evaluation focused on pragmatic assessment of sustained implementation rather than causal inference.

Statistical analyses were performed using Stata version 17 (StataCorp LLC, College Station, TX, USA), with a two‐sided *p*‐value < 0.05 considered statistically significant.

### 2.7 Ethical Considerations

This quality improvement initiative was reviewed and approved by the institutional review board (DSRB reference number 2024/3433; approval granted on 20 August 2024). A waiver of informed consent was granted because the project involved secondary analysis of de‐identified data collected as part of routine clinical care and quality monitoring. Patient‐level data were extracted from the electronic medical record in de‐identified form and analysed in aggregate. Staff participation in the survey and interviews was voluntary, responses were anonymised, and findings were reported only at the group level. The initiative was conducted in accordance with institutional requirements for quality improvement and principles for the ethical use of routinely collected healthcare data.

## 3 Results

### 3.1 Study Population and Oxygen Modalities

During the study period, 726 patients contributed 8307 patient‐days of supplemental oxygen therapy, including 2686 patient‐days in the pre‐intervention phase and 5621 in the post‐intervention phase (Table 1).

**TABLE 1:** Distribution of patient‐days by oxygen modality and study phase, *n* (%).

Oxygen modality	Pre‐intervention (Apr to September 2024) [*n* = 255, 2686 patient‐days]	Post‐intervention (January to December 2025) [*n* = 471, 5621 patient‐days]	Total [*n* = 726, 8307 patient‐days]
Conventional oxygen, including room air	1782 (66.3%)	4025 (71.6%)	5807 (69.9%)
Non‐invasive ventilation (NIV)	115 (4.3%)	240 (4.3%)	355 (4.3%)
Mechanical ventilation (MV)	661 (24.6%)	1048 (18.6%)	1709 (20.6%)
High‐flow nasal cannula (HFNC)	128 (4.8%)	308 (5.5%)	436 (5.2%)

*Note:* Percentages calculated using patient‐days per phase.

### 3.2 Primary Process Measure: Oxygen Compliance

**FIGURE 1:**
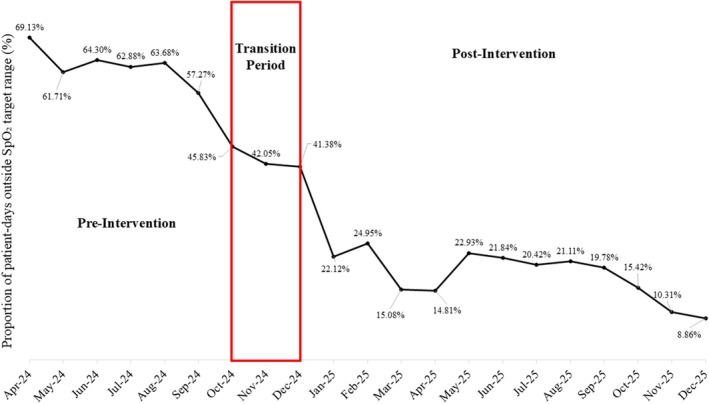
Monthly proportion of patient‐days outside the target SpO_2_ range across the study period (*n* = 8307 patient‐days).

The proportion of patient‐days outside the prescribed SpO_2_ target range decreased from a median of 63% (IQR 57–65) in the pre‐intervention phase to 20% (IQR 15–22) following implementation. Improvements were sustained throughout the 12‐month post‐intervention period, with progressive reductions over time, reaching below 10% by December 2025 (Figure 1).

### 3.3 Oxygen Consumption Outcomes

**FIGURE 2:**
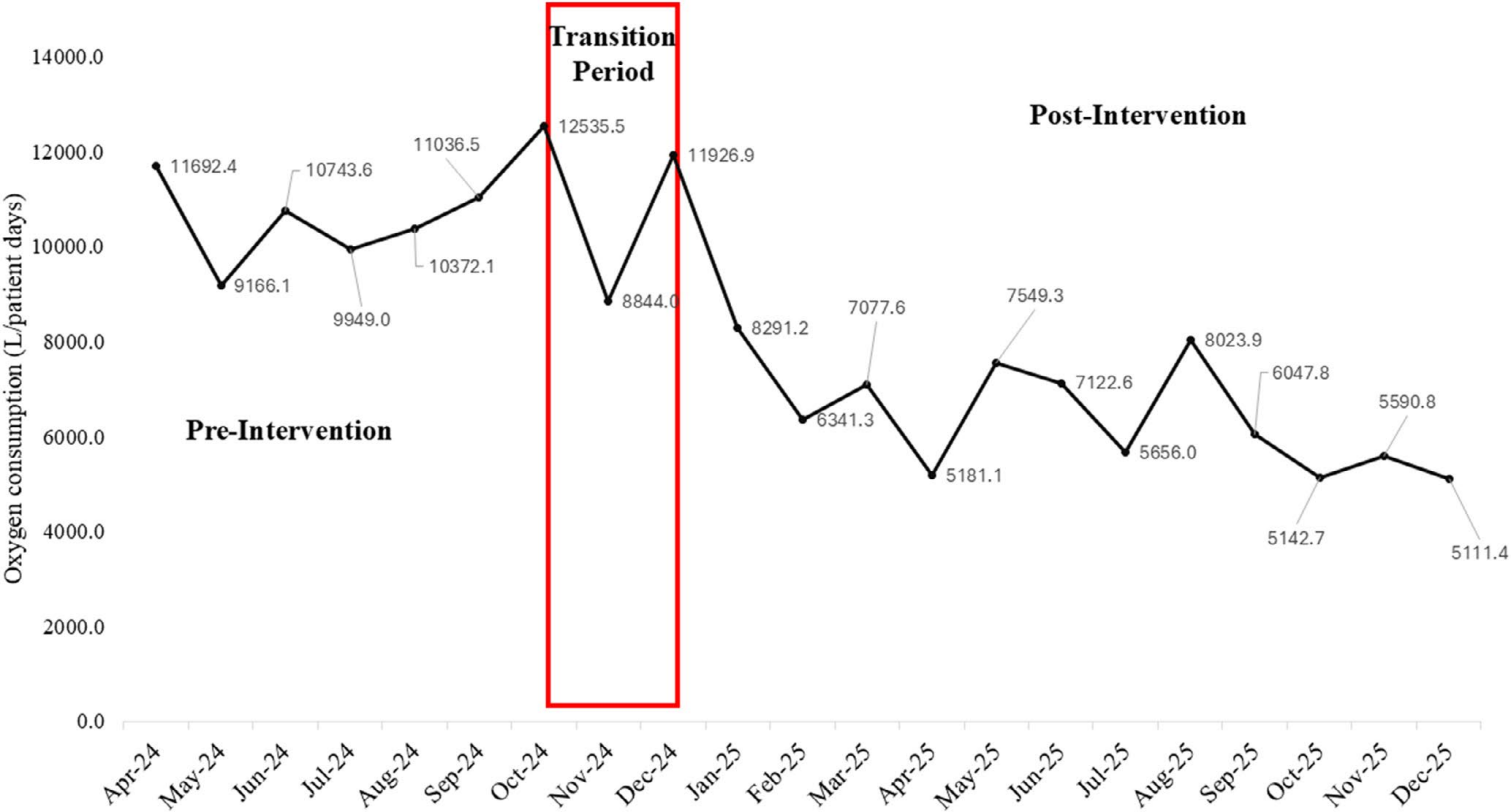
Monthly total oxygen consumption per patient‐day across all oxygen delivery modalities during the pre‐intervention, transition and post‐intervention periods (*n* = 8307 patient‐days).

Total oxygen consumption per patient‐day declined following implementation of the oxygen stewardship initiative across all delivery modalities. Monthly trends demonstrated sustained reductions during the post‐intervention period (Figure 2).

**FIGURE 3:**
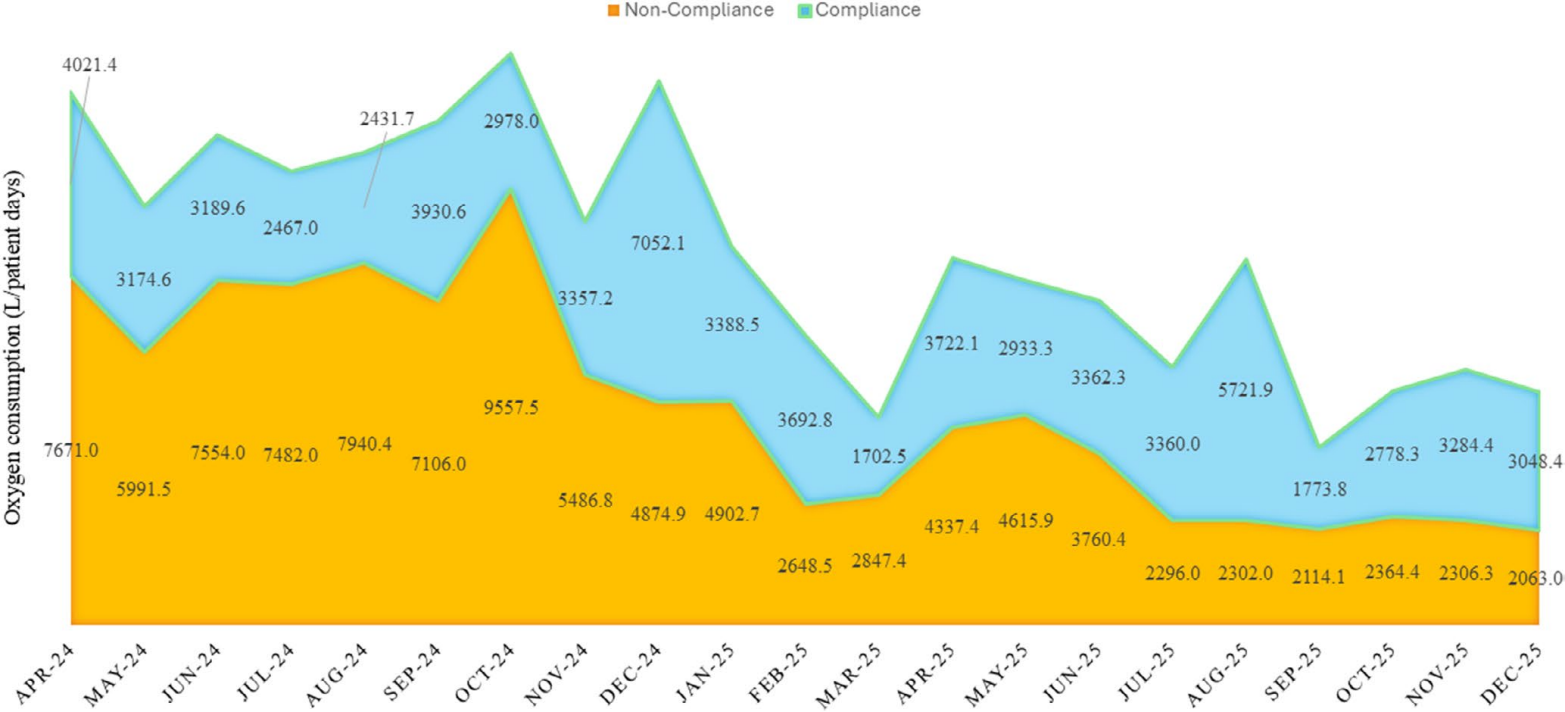
Monthly oxygen consumption per patient‐day stratified by compliance with recommended oxygen saturation targets (*n* = 8307 patient‐days).

Reductions in total oxygen consumption were primarily driven by decreased oxygen use during non‐compliant periods, while oxygen use within target saturation ranges remained comparatively stable (Figure 3).

At the modality level, median monthly oxygen consumption per patient‐day decreased significantly for conventional oxygen therapy, non‐invasive ventilation and mechanical ventilation (Table 2). Although HFNC therapy demonstrated a numerical reduction, this change did not reach statistical significance.

**TABLE 2:** Median monthly oxygen consumption per patient‐day by modality before and after intervention.

Oxygen modality	Pre‐intervention median (IQR), L/patient‐day	Post‐intervention median (IQR), L/patient‐day	*p*
Conventional oxygen therapy	3600 (3170–4030)	1870 (1300–2590)	< 0.001*
Non‐invasive ventilation	5810 (5110‐6240)	3540 (2250‐4760)	0.003*
Mechanical ventilation	8610 (7630‐9220)	4650 (3300‐6610)	< 0.001*
High‐flow nasal cannula	28 800 (18800‐34 600)	21 600 (17500‐32 900)	0.098

*Note:* Pre‐ intervention period: April–September 2024. Post‐ intervention period: January–December 2025. Monthly oxygen consumption values were normalised per patient‐day and summarised as medians (IQR) across months. Transition months (October to December 2024) were excluded a priori. *p < 0.05.

Based on the observed reduction in oxygen consumption per patient‐day, the stewardship intervention corresponded to an estimated reduction of approximately 14 500 m^3^ of medical oxygen during the 12‐month post‐intervention period (Figure S1). Using approximate internal institutional estimates of medical oxygen supply costs, this reduction may correspond to around SGD 9000–10 000 in avoided oxygen expenditure annually, although a formal economic evaluation was beyond the scope of this study.

No increase in clinically significant hypoxia or adverse respiratory events was identified during the post‐intervention period based on routine ICU incident reporting and clinical monitoring. Formal safety endpoints, such as desaturation events or escalations in respiratory support, were not systematically collected as part of this quality improvement evaluation.

A summary of Plan‐Do‐Study‐Act (PDSA) cycles and key refinements is provided in Supplementary Table S2. Findings from the anonymous cross‐sectional staff survey and semi‐structured interviews informed iterative adjustments to educational messaging, protocol clarification and reinforcement of documentation practices (Figure S2).

### 3.4 Process Evaluation: Staff Survey Findings

A total of 131 ICU staff participated in the survey, including 95 nurses (72.5%), 21 respiratory therapists (16.0%) and 15 physicians (11.5%). The median ICU experience was 5 years (IQR 1–10). Staff who attended the in‐service education (*n* = 74) reported greater confidence in titrating oxygen to target saturation ranges compared with non‐attendees (*n* = 59) (median confidence score 4 [IQR 3–5] vs. 3 [IQR 2–4], *p* = 0.018).

Key clinical factors guiding oxygen weaning decisions included oxygen saturation stability, work of breathing, respiratory rate, arterial blood gas results and patient comfort. Commonly reported barriers included concerns regarding patient deterioration and safety when reducing oxygen therapy, whereas enabling factors included the presence of a standardised weaning protocol and clear documentation of oxygen saturation targets (Table 3).

**TABLE 3:** Staff survey responses related to oxygen weaning practices (*n* = 131).

Survey statement	Strongly disagree	Disagree	Neutral	Agree	Strongly agree
I am confident in identifying appropriate SpO_2_ targets	0 (0.0%)	2 (1.5%)	11 (8.4%)	53 (40.5%)	65 (49.6%)
Reducing unnecessary oxygen use is important	3 (2.3%)	3 (2.3%)	21 (16.0%)	47 (35.9%)	57 (43.5%)
Concern about deterioration makes me hesitant to wean from oxygen	6 (4.6%)	15 (11.5%)	39 (29.8%)	45 (34.4%)	26 (19.8%)
Clear documentation supports oxygen weaning	1 (0.8%)	2 (1.5%)	12 (9.2%)	31 (23.7%)	85 (64.9%)

*Note:* Values are presented as *n* (%). Percentages are calculated based on the total number of respondents (*n* = 131).

Table 4 summarises key themes from semi‐structured interviews exploring contextual and behavioural factors influencing oxygen weaning practices.

**TABLE 4:** Key themes identified from individual interviews during process evaluation.

Theme	Description	Example quote
Patient safety concerns	Clinicians expressed caution about reducing oxygen due to fear of deterioration	‘We tend to keep oxygen slightly higher because we worry the patient may desaturate unexpectedly’.
HFNC uncertainty	Greater hesitation in HFNC weaning	‘HFNC feels safer, so we are sometimes slower to reduce oxygen’.
Patient comfort	Anxiety influenced decisions	‘Some patients feel breathless when oxygen is reduced, even if saturation is acceptable’.
Workload pressures	Time constraints limited reassessment	‘During busy shifts oxygen is sometimes left unchanged’.
Documentation gaps	Lack of clear targets across shifts	‘If the target SpO_2_ is not written clearly, different staff may manage oxygen differently’.

## 4 Discussion

This quality improvement evaluation demonstrates that a structured oxygen stewardship programme was associated with sustained improvements in oxygen titration practice and reductions in overall oxygen consumption in a tertiary adult ICU. Importantly, reductions in total oxygen use were primarily driven by decreased oxygen delivery during non‐compliant periods, while oxygen use within target ranges remained stable. This suggests that improvements were achieved by correcting excessive practice rather than restricting clinically appropriate therapy.

Compliance improved progressively across successive quality improvement cycles. Initial gains followed the implementation of a standardised oxygen weaning protocol and multidisciplinary education, consistent with evidence that guideline dissemination alone produces modest improvements [12]. Subsequent incorporation of audit feedback and real‐time reinforcement into routine workflows was associated with further stabilisation and sustainability of compliance [10, 11, 12]. These findings align with implementation science evidence that active feedback and workflow integration are more effective than passive dissemination strategies in promoting sustained adherence.

A higher proportion of patient‐days outside target ranges was observed among patients receiving HFNC therapy. Interview findings highlighted uncertainty about HFNC weaning thresholds and perceived safety margins for high‐flow therapy. Modalities perceived as safer may inadvertently encourage more liberal oxygen use [13]. These findings suggest that modality‐specific guidance and clearer escalation criteria may be necessary to optimise oxygen stewardship efforts and reduce persistent variation in practice.

Although the primary focus of this evaluation was clinical practice improvement, reductions in oxygen consumption may also have broader sustainability implications. Using published conversion factors for medical oxygen (0.0003–0.0021 kg CO_2_e/L), the observed reduction in oxygen consumption corresponds to an estimated 1.0–6.8 kg CO_2_e per patient‐day and approximately 12–84 t CO_2_e annually for a 34‐bed ICU operating at full occupancy [14, 15]. Although direct environmental measurement was beyond the scope of this evaluation, these estimates contextualise the potential environmental implications of oxygen stewardship within broader healthcare sustainability efforts.

In addition to environmental benefits, reductions in oxygen consumption may also have economic implications. As reported in the Results, the stewardship intervention was associated with measurable reductions in overall oxygen use. Although a formal cost analysis was beyond the scope of this evaluation, reduced oxygen utilisation may translate into modest reductions in oxygen procurement costs. The magnitude of these savings will depend on local supply infrastructure and procurement contracts.

These results align with international recommendations identifying appropriate oxygen management as a core component of essential emergency and critical care. Previous studies have demonstrated that education, standardisation and system‐level prompts can reduce unnecessary oxygen exposure. This study extends existing work by demonstrating how a clinician‐led, context‐specific quality improvement approach can translate guideline‐recommended targets into sustained bedside practice within routine ICU workflows.

Beyond reductions in oxygen consumption, the stewardship programme appeared to influence clinician behaviour and shared accountability. Embedding oxygen targets into handovers, documentation processes and reinforcement mechanisms shifted oxygen management from passive continuation to active titration. Integrating oxygen review into the routine nursing workflow promoted greater ownership of oxygen prescribing and weaning decisions across professional groups.

Although oxygen stewardship may plausibly reduce workflow inefficiencies and unnecessary monitoring adjustments, this evaluation did not formally assess clinician workload, staffing utilisation or cognitive burden. Oxygen stewardship may represent a practical and nurse‐led strategy to enhance the reliability of oxygen titration while supporting responsible resource use in critical care. Future studies incorporating validated workload or human‐factors measures may help clarify the broader systems impact of stewardship interventions.

## 5 Limitations

Several limitations should be acknowledged. This quality improvement evaluation did not assess downstream clinical outcomes such as ICU length of stay or mortality. The study was not designed or powered to evaluate these endpoints, and the before‐and‐after design without adjustment for illness severity or case‐mix limits causal inference.

Oxygen consumption estimates were derived from routinely documented clinical data and relied on time‐weighted assumptions rather than continuous high‐resolution measurements. While this pragmatic approach reflects real‐world quality monitoring, measurement imprecision cannot be excluded.

Although secular trends may have contributed to observed improvements, no concurrent institutional initiatives specifically targeting oxygen use were implemented during the study period. Finally, this evaluation was conducted in a single tertiary ICU, which may limit generalisability. However, the intervention utilised low‐cost, workflow‐integrated strategies that may be adaptable to other acute care settings.

## 6 Implications for Clinical Practice

This study demonstrates that structured oxygen stewardship can be successfully integrated into routine critical care practice. Embedding oxygen targets into nursing workflows, documentation processes and handovers supported consistent titration behaviour and reduced unnecessary oxygen use. Nurse‐engaged stewardship initiatives may strengthen accountability for oxygen therapy management while promoting adherence to guideline‐recommended targets. Similar low‐cost, workflow‐integrated interventions may be feasible in other intensive care settings.

## 7 Conclusion

Oxygen stewardship can be embedded into routine ICU workflows and sustained over time, resulting in improved adherence to target oxygen saturation ranges and reduced unnecessary oxygen use. Integrating a standardised weaning protocol with multidisciplinary education and real‐time feedback supported consistent bedside titration without restricting appropriate therapy. This pragmatic, low‐cost approach may be transferable to other acute care settings. Future work should focus on modality‐specific optimisation, particularly for high‐flow nasal cannula therapy, and on scalable decision‐support strategies to strengthen oxygen stewardship.

## Author Contributions

**Wei Jun Dan Ong:** conceptualisation, methodology, investigation, formal analysis, data curation, project administration, visualisation, writing – original draft. **Glen JiaXin Li:** methodology, data curation, writing – review and editing. **Lawrence Matunan Ace Azul:** conceptualisation, resources, supervision, writing – review and editing. **Anna Kristina Trajico:** investigation, data curation, writing – review and editing. **Wee Kian Ricardo Tan:** investigation, writing – review and editing. **Jared D'Souza:** supervision, validation, writing – review and editing. **Faheem Ahmed Khan:** conceptualisation, methodology, supervision, validation, writing – review and editing. **Amit Kansal:** supervision, conceptualisation, methodology, formal analysis, validation, writing – original draft. All authors reviewed and approved the final manuscript.

## Funding

The authors have nothing to report.

## Ethics Statement

This study was reviewed and approved by the Institutional Review Board of Ng Teng Fong General Hospital (NUHS), Singapore. National Healthcare Group Domain‐Specific Review Board (NHG DSRB Ref: 2024/3433; approval on 20th August 2024). The study was performed in accordance with the principles of the Declaration of Helsinki and applicable local regulations.

## Consent

The authors have nothing to report.

## Conflicts of Interest

The authors declare no conflicts of interest.

## Supporting information


**Figure S1:** Trends in oxygen non‐compliance by delivery modality over time.
**Figure S2:** Examples of implementation artefacts supporting oxygen stewardship in the ICU.
**Table S1:** Device‐specific oxygen consumption estimation formulas.
**Table S2:** Iterative Plan‐Do‐Study‐Act (PDSA) cycles for implementation of the oxygen stewardship intervention.

## Data Availability

The data that support the findings of this study are available on request from the corresponding author. The data are not publicly available due to privacy or ethical restrictions.
