# Supplementary material for: Oxygen Stewardship in Adult Critical Care: A Quality Improvement Initiative
Source: Nurs Crit Care. 2026 Apr 6;31(3):e70476. doi: 10.1111/nicc.70476 (PMC13051414; doi:10.1111/nicc.70476)
Supplement: Supplementary file 1 — Figure S1: Trends in oxygen non‐compliance by delivery modality over time. Figure S2: Examples of implementation artefacts supporting oxygen stewardship in the ICU. Table S1: Device‐specific oxygen consumption estimation formulas. Table S2: Iterative Plan‐Do‐Study‐Act (PDSA) cycles for implementation of the oxygen stewardship intervention. [file NICC-31-0-s001.docx]

**Supplementary Materials**

**Figure S1. Trends in oxygen non-compliance by delivery modality over time.**

The figure illustrates monthly trends in the proportion of patient-days exceeding recommended oxygen saturation targets, stratified by oxygen delivery modality - conventional oxygen therapy, non-invasive ventilation (NIV), mechanical ventilation (MV), and high-flow nasal cannula (HFNC). Early reductions in non-compliance coincided with implementation of the standardized oxygen weaning protocol and multidisciplinary education, followed by further stabilization during later phases incorporating spot-check feedback and iterative refinement.

Data beyond the primary study period were collected as part of routine post-implementation monitoring to assess the stability of practice change and were not included in formal statistical analyses. This figure is provided for transparency and quality improvement learning rather than outcome evaluation.


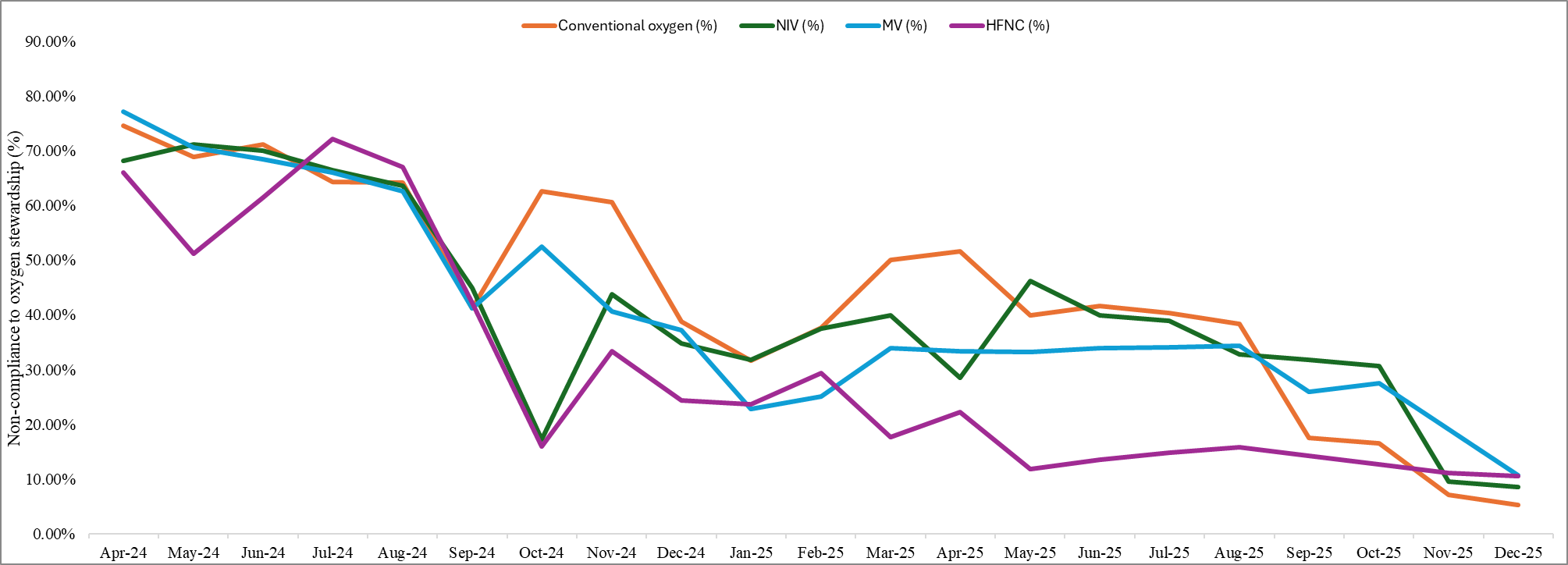


**Figure S2. Examples of implementation artefacts supporting oxygen stewardship in the ICU.**

| A |  |
| --- | --- |
| 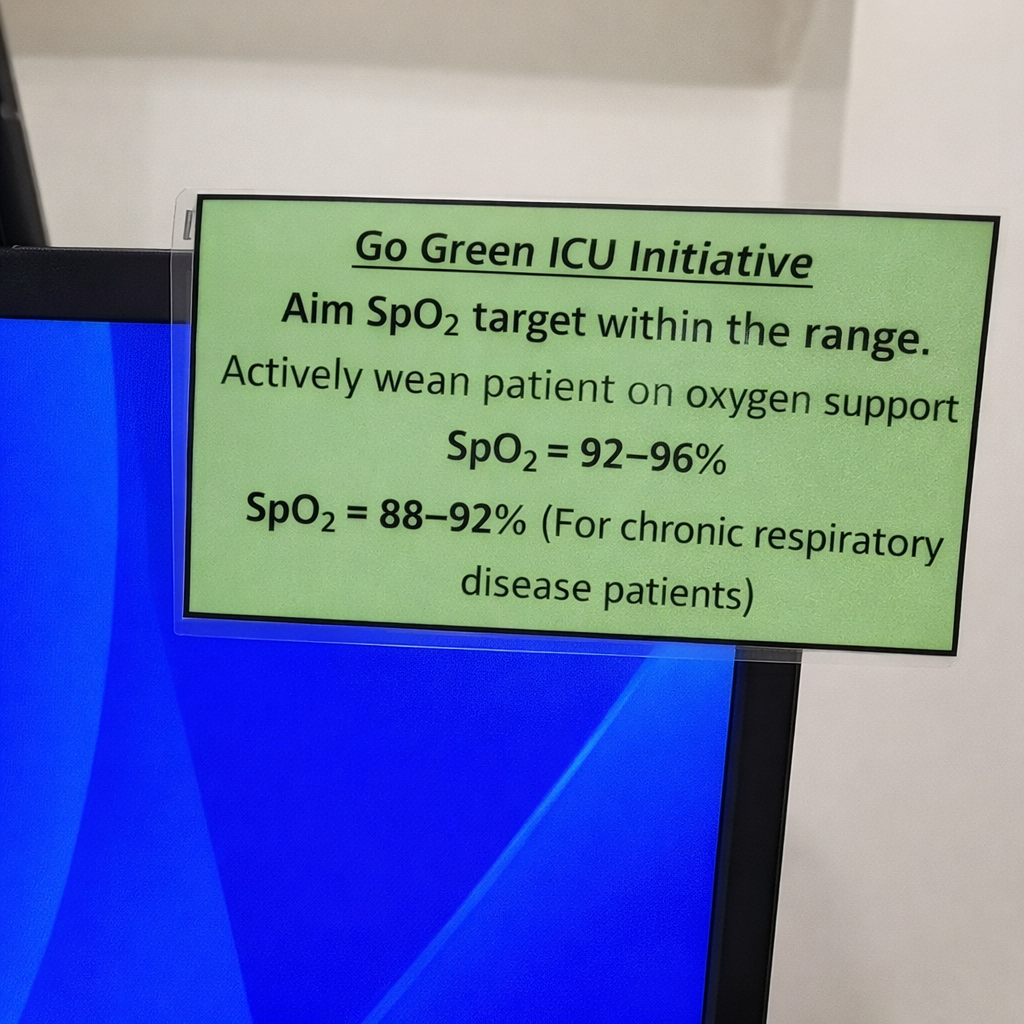 |  |
| B | |
| 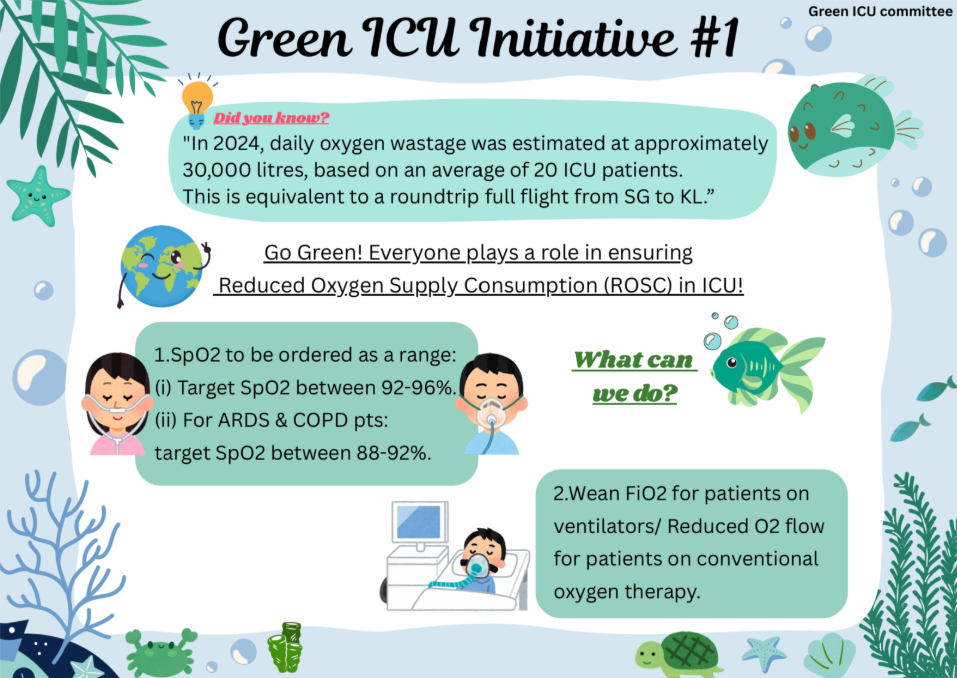 | |
| C | |
| 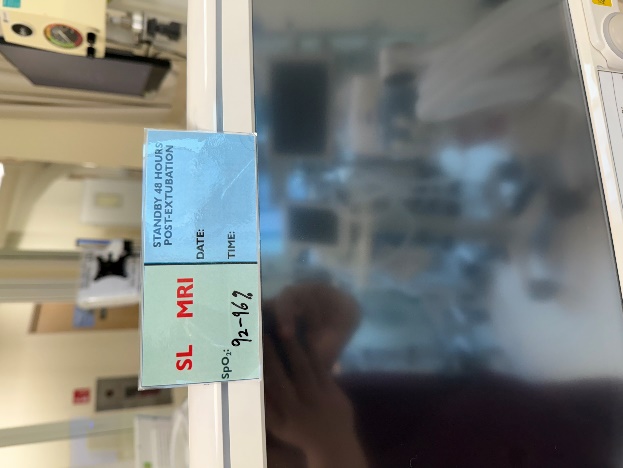 | |

Panel A shows a bedside visual reminder displaying recommended oxygen saturation targets at a clinical workstation. Panel B illustrates a unit-level quality improvement display that reinforces oxygen stewardship messaging and staff engagement. Panel C shows a bedside oxygen target label placed on monitoring equipment to prompt documentation and titration within recommended ranges. These artefacts were used to support protocol adherence and were not evaluated as an independent intervention.

**Table S1. Device-specific oxygen consumption estimation formulas**

Oxygen consumption was estimated using modality-specific formulas based on routinely documented clinical parameters to support comparative analysis across study phases, rather than precise measurement of device oxygen uptake.

| **Oxygen modality** | **Oxygen consumption estimation formula** | **Key assumptions/notes** |
| --- | --- | --- |
| Conventional oxygen therapy (COT) | Oxygen volume (L) = Flow rate (L/min) × Duration (min) | Assumes continuous flow at the documented setting. |
| Non-invasive ventilation (NIV) | Oxygen volume (L) = [(Estimated total flow demand + Estimated leak flow) × FiO₂] × Duration (min) | Accounts for intentional and unintentional leak. Leak flow is estimated from the ventilator-reported leak or assumed proportional to pressure support and interface type. |
| Mechanical ventilation (MV) | Oxygen volume (L) = [(Minute ventilation + Bias flow) × FiO₂] × Duration (min) | Bias flow represents a continuous base flow intrinsic to the ventilator mode and the device. The formula estimates the effective delivered oxygen rather than the direct device uptake. |
| High-flow nasal cannula (HFNC) | Oxygen volume (L) = Total flow (L/min) × FiO₂ × Duration (min) | Total flow represents blended gas flow. Oxygen fraction determined by set FiO₂. Assumes stable flow during documented duration. |

**Definitions**

- **Minute ventilation** = Tidal volume × Respiratory rate
- **Bias flow** = Continuous base flow generated by the ventilator during expiration (mode and device-dependent)
- **Estimated total flow demand (NIV)** = Inspiratory flow required to meet patient demand under pressure support
- **Estimated leak flow (NIV)** = Excess flow required to maintain target pressure in the presence of interface leak
- **FiO₂** = Fraction of inspired oxygen
- **Duration** = Time on therapy (minutes)

**Table S2. Iterative Plan-Do-Study-Act (PDSA) cycles for implementation of the oxygen stewardship intervention**

The table outlines the sequential testing of intervention components, including protocol standardization, staff education, real-time reinforcement through spot checks, and process evaluation using staff surveys and semi-structured individual interviews. Each cycle documents the planned intervention, implementation approach, observations derived from quantitative monitoring and qualitative feedback, and subsequent refinements.

| **PDSA cycle** | **Plan** | **Do** | **Study** | **Act** |
| --- | --- | --- | --- | --- |
| **Cycle 1: Protocol introduction and education** | Introduce a standardised oxygen weaning protocol aligned with guideline-recommended SpO₂ targets and increase baseline staff awareness of oxygen stewardship. | Conducted a baseline staff survey to assess clinicians’ knowledge, perceived barriers, and attitudes toward oxygen weaning and oxygen stewardship, alongside unit-wide in-service education sessions covering target SpO₂ ranges, risks of hyperoxia, and principles of oxygen titration across delivery modalities. | Survey findings and early review showed variable baseline understanding, inconsistent documentation of oxygen targets, and uncertainty around oxygen weaning in selected clinical scenarios despite improved conceptual awareness following education. | Refined protocol emphasis on documentation of target SpO₂ ranges and escalation criteria; introduced visual reminders at bedside workstations. |
| **Cycle 2: Real-time reinforcement through spot checks** | Reinforce adherence by identifying and addressing deviations from target oxygen saturation ranges during routine care. | Performed periodic spot checks of SpO₂ values and corresponding oxygen settings, with immediate feedback to bedside teams when patient-days fell outside target ranges. | Spot checks demonstrated improved compliance for conventional oxygen therapy and mechanical ventilation, with persistent liberal oxygen use in selected modalities. | Focused feedback on high-risk scenarios and identified the need for more sustained reinforcement beyond episodic spot checks. |
| **Cycle 3: Sustained reinforcement through constant reminders** | \|  \| \| --- \|  \| Sustain improvements in oxygen compliance by embedding constant reminders into routine ICU workflows. \| \| --- \| | Implemented constant visual and verbal reminders of oxygen targets at the bedside, incorporated oxygen targets into standard handover templates, and delivered weekly reinforcement messages during multidisciplinary briefings. | Ongoing monitoring demonstrated sustained improvements in oxygen compliance for conventional oxygen therapy and mechanical ventilation, with reduced day-to-day variability in adherence. | Refined reminder content to prioritise high-risk scenarios and simplified messaging to minimise cognitive burden and support routine uptake. |
| **Cycle 4: Exploration of behavioural and contextual barriers** | Identify human-factors and contextual contributors to persistent variation in adherence to oxygen targets. | Conducted semi-structured individual interviews with multidisciplinary ICU staff to explore decision-making processes, perceived risks, and barriers to oxygen weaning. | Key themes included concern about patient deterioration, uncertainty around safe weaning thresholds (particularly for HFNC), workload pressures, and inconsistent communication of oxygen targets. | Informed targeted refinements, including clearer HFNC guidance, reinforcement of shared mental models, and emphasis on documenting oxygen targets during ward rounds. |
| **Cycle 5: Consolidation and sustainability** | Embed oxygen stewardship into routine ICU workflows and promote sustainability of practice change. | Integrated oxygen target documentation and weaning prompts into routine clinical processes, supported by ongoing education and feedback loops. | Improvements in compliance were sustained over time and accompanied by reductions in overall oxygen consumption at the unit level. | Maintained intervention as standard practice, with plans for further modality-specific refinement and exploration of digital decision support tools. |

*PDSA cycles were implemented sequentially during the transition and early post-intervention phases and were refined iteratively based on ongoing monitoring.*
